# Post-translational modulation of cell signalling through protein succinylation

**DOI:** 10.37349/etat.2023.00196

**Published:** 2023-12-27

**Authors:** Katharina F. Kubatzky, Yue Gao, Dayoung Yu

**Affiliations:** Istituto Nazionale Tumori-IRCCS-Fondazione G. Pascale, Italy; ^1^Department of Infectious Diseases, Medical Faculty Heidelberg, Medical Microbiology and Hygiene, Heidelberg University, 69120 Heidelberg, Germany; ^2^Department of Infectious Diseases, University Hospital Heidelberg, 69120 Heidelberg, Germany

**Keywords:** Succinate, metabolites, mitochondria, post-translational modifications, lysine succinylation, cancer, immune system

## Abstract

Cells need to adapt their activities to extra- and intracellular signalling cues. To translate a received extracellular signal, cells have specific receptors that transmit the signal to downstream proteins so that it can reach the nucleus to initiate or repress gene transcription. Post-translational modifications (PTMs) of proteins are reversible or irreversible chemical modifications that help to further modulate protein activity. The most commonly observed PTMs are the phosphorylation of serine, threonine, and tyrosine residues, followed by acetylation, glycosylation, and amidation. In addition to PTMs that involve the modification of a certain amino acid (phosphorylation, hydrophobic groups for membrane localisation, or chemical groups like acylation), or the conjugation of peptides (SUMOylation, NEDDylation), structural changes such as the formation of disulphide bridge, protein cleavage or splicing can also be classified as PTMs. Recently, it was discovered that metabolites from the tricarboxylic acid (TCA) cycle are not only intermediates that support cellular metabolism but can also modify lysine residues. This has been shown for acetate, succinate, and lactate, among others. Due to the importance of mitochondria for the overall fitness of organisms, the regulatory function of such PTMs is critical for protection from aging, neurodegeneration, or cardiovascular disease. Cancer cells and activated immune cells display a phenotype of accelerated metabolic activity known as the Warburg effect. This metabolic state is characterised by enhanced glycolysis, the use of the pentose phosphate pathway as well as a disruption of the TCA cycle, ultimately causing the accumulation of metabolites like citrate, succinate, and malate. Succinate can then serve as a signalling molecule by directly interacting with proteins, by binding to its G protein-coupled receptor 91 (GPR91) and by post-translationally modifying proteins through succinylation of lysine residues, respectively. This review is focus on the process of protein succinylation and its importance in health and disease.

## Introduction

Post-translational modifications (PTMs) regulate protein activity by modifying specific amino acid residues resulting in reversible or irreversible chemical modifications of the protein [1]. PTMs can for example change the catalytic activity, modify the interaction with other proteins, or change the subcellular localisation. More than 300 different types of PTMs have been reported, but phosphorylation, acetylation, and glycosylation are the most frequently found and best-characterised PTMs [2].

Metabolic activity is a hallmark of a living cell. Changes in activity to accommodate increased proliferation or cellular activation go along with an alteration in metabolic activity and the prevalence of certain metabolites. Metabolites are increasingly getting recognised for their ability to modify protein signalling, especially through the modification of lysine residues [3]. Lysine is a positively charged essential amino acid and its modification causes a major change in protein structure due to the replacement of the positive charge. Lysine acetylation of histones was already detected in 1964, but the general concept of lysine modification came into the focus of research much later, with better mass spec analysis methods being available [4–6]. In addition to lactylation, malonylation, succinylation, glutarylation, propionylation, and butyrylation of lysine residues have been described [7–11]. Not quite unexpected, metabolite-type PTMs are often involved in the regulation of metabolic pathways and mitochondrial proteins [12]. However, as observed for acetylation, histone modifications and associated epigenetic changes are also observed for other lysine acylations [13].

Already in the 18th century, French scientist Antoine Lavoisier demonstrated that living organisms consume oxygen to be able to degrade “fuels” to release energy [14]. In 1856, Louis Pasteur laid the basis for the understanding of cellular metabolism by his discovery that yeast depletes glucose from the medium while there in an increase in CO_2_ and lactate [14]. Otto Warburg finally made the ground-braking discovery that also mammalian cells can “ferment” glucose in the presence of oxygen, a metabolic process termed “aerobic glycolysis” [14]. In 1931 he received the Nobel Prize for his observation that rapidly proliferating cancer cells preferred glycolysis to generate energy, despite the presence of oxygen. In the glycolytic pathway, glucose uptake results in glycolytic activity and the production of pyruvate, which is further reduced to lactate. By this, glycolytic flux is maintained as nicotinamide adenine dinucleotide (NAD^+^) gets reduced to the reduced form of NAD (NADH) at the same time. In addition, glycolytic intermediates can serve as building blocks for anabolic activities and the generation of ribose or fatty acids. This compensates for the reduced levels of adenosine triphosphate (ATP) that can be generated from glycolysis. Many studies have highlighted that in cancer, cellular metabolism is drastically changed due to an increased need for glucose. Interestingly, many of these observations made earlier in cancer research can be transferred to activated immune cells, especially innate immune cells like macrophages and dendritic cells (DCs), where a change in activity is accompanied by a detectable shift in the abundance of certain metabolites [15]. Research on immune cell metabolism shows that quiescent or naive cells prefer ATP generation via oxidative phosphorylation (OxPhos) using the Krebs cycle, while activated cells utilise glycolysis and exhibit anabolic activity. This allows them to rapidly build up pro-inflammatory mediators and fight infections [15].

**Figure 1:**
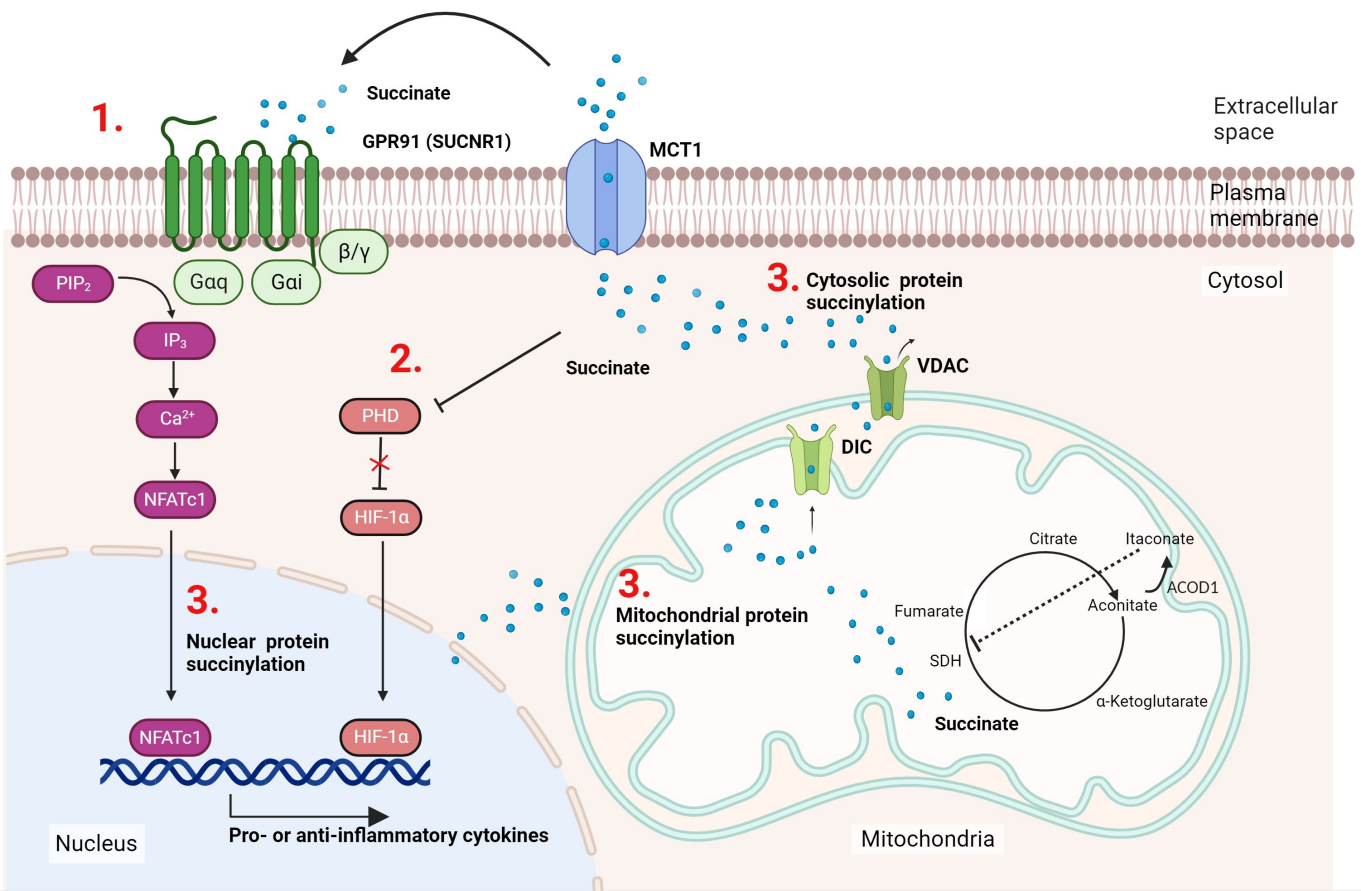
Succinate accumulation facilitates actions in immune cells. M1 macrophages generate itaconate through cis-aconitate decarboxylase (ACOD1) induction, this blocks SDH and causes succinate accumulation in the mitochondria. Succinate can be released from the mitochondria or the cell, respectively, via the transporters mitochondrial dicarboxylate carrier (DIC), voltage-dependent anion channel (VDAC), or monocarboxylate transporter (MTC1) and participate in cell signalling processes via the following three mechanisms: (1) after export to the extracellular space it can bind as the ligand to its cognate receptor GPR91 activating typical G protein receptor signalling pathways via Galphai (Gαi) and Galphaq (Gαq); (2) succinate can mediate HIF-1α stabilisation, ultimately supporting IL-1β production; (3) attachment of succinate via non-enzymatic or enzymatic reactions occur in the mitochondria, cytosol, and the nucleus. SUCNR1: succinate receptor-1; NFATc1: nuclear factor of activated T-cells 1; PIP_2_: phosphatidylinositol 4,5-bisphosphate; IP_3_: inositol-1,4,5-trisphosphate. This figure was created by Biorender.com

Studies in the 1960ies had already noted the requirement of increased glucose levels for activated immune cells [16–18], but only the development of new experimental tools in the 21st century made more detailed investigations possible. The last twenty years have seen a tremendous increase in knowledge especially for T cells, DCs, and macrophages, but also B cells or even neutrophils show a similar connection between their respective functional phenotypes and metabolic pathways [19, 20]. While the anti-inflammatory M2 macrophage is characterised by primarily using OxPhos, hypoxic conditions or stimulation through pattern recognition receptors results in a Warburg-type shift towards glycolysis and fatty acid oxidation [15]. Succinate accumulation was found to be mainly caused by glutamine-dependent anaplerosis and the gamma-aminobutyric acid (GABA) shunt pathway, but disruption of the tricarboxylic acid (TCA) cycle by itaconate-mediated inhibition of succinate dehydrogenase (SDH) contributes to this effect [15]. This causes the accumulation of succinate, which plays a role in immune cell activity through various processes (Figure 1). As shown in the section labeled 1 in Figure 1, succinate can bind extracellularly as a ligand to the G protein-coupled receptor 91 (GPR91) and activate it, which can subsequently support both pro-inflammatory and anti-inflammatory processes [21, 22]. Section 2 highlights the fact that intracellular succinate can inhibit the enzyme prolyl hydroxylase (PHD) at high concentrations [23]. As PHD produces succinate as a by-product when the enzyme hydroxylates hypoxia-inducible factor 1alpha (HIF-1α) to HIF-1α-OH, accumulation of succinate inhibits PHD activity by product inhibition. This results in the stabilisation of HIF-1α, a phenomenon termed pseudohypoxia. As a consequence of HIF-1α activation, pro-inflammatory interleukin-1beta (IL-1β) is produced. Section 3 in Figure 1 refers to the use of intracellular succinate for lysine succinylation. This process occurs primarily in the mitochondria, but modified cytoplasmic proteins and succinylated histones have also been identified [24]. In this review, the effects of protein succinylation in cancer and immune-related diseases are summarised and the connections of the various succinate-mediated pathways are discussed.

## Mechanisms of protein succinylation

**Figure 2:**
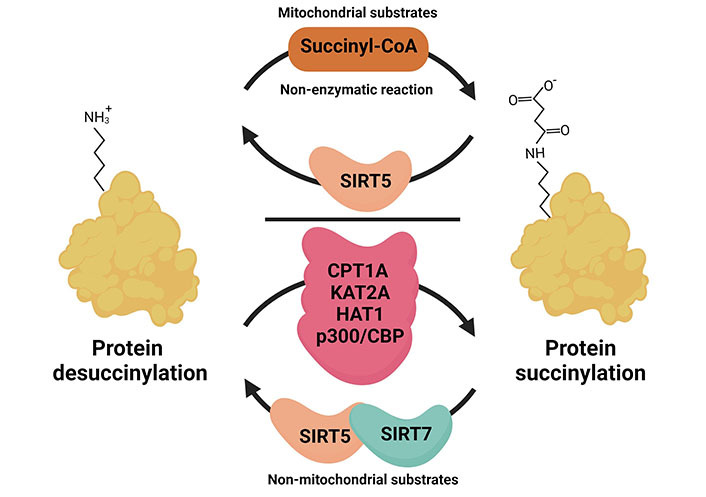
Mechanism of succinylation in different subcellular compartments. The addition of succinyl occurs non-enzymatically in the mitochondria where the intermediate metabolite succinyl-CoA is abundantly produced. The removal of succinylation from the protein requires desuccinylase SIRT5. In the cytoplasm and nucleus, the reactions of succinylation and desuccinylation often require enzymatic action. For example, HAT1 and CPT1A involved as cytoplasmic succinyltransferases, KAT2A, HAT1, and p300/CBP were found to have catalytic activity in the nucleus and support histone succinylation. SIRT5 and SIRT7, both can act as nuclear desuccinylases. This figure was created by Biorender.com

A new type of PTM involves the attachment of metabolites. PTMs affect the localisation, interaction, stability, and conformational changes of proteins. Thus, metabolic cues can directly influence the signalling activities of the proteins they attach to [3]. Artificial succinylation of proteins was already investigated in the 1960ies and is for example used to modify anti-neoplastic drugs. Due to the drastic changes in protein net charges succinylated drugs may show decreased renal accumulation and increased blood clearance which can be beneficial for the patient [25, 26]. Lysine succinylation was discovered as a naturally occurring PTM in 2011 [9]. A shift in mass of 100,0186 Da was detected in a mass spectrometry (MS) approach that profiled peptides derived from the tryptic digestion of *Escherichia* (*E.*) *coli* isocitrate dehydrogenase (IDH). As observed for other lysine modifications, lysine succinylation was found to be evolutionary conserved in pro- and eukaryotic organisms [9]. It was suggested that a non-enzymatic mechanism is the prevalent mode of action, where the concentration of the reactant, succinyl-coenzyme A (CoA), is the driving force for attachment (Figure 2) [24]. Succinyl-CoA can be derived from amino acid metabolism or the TCA cycle. This hypothesis was supported by the finding that many succinylated proteins are localised in the mitochondria where the succinate concentration is highest. Succinate, however, can also be exported from the mitochondria using solute carrier (Slc) transporters, and get converted from cytosolic succinate to succinyl-CoA again, thus allowing its attachment to cytoplasmic or nuclear proteins. More recently, four enzymes were independently identified that can succinylate proteins as an additional non-canonical function, namely carnitine palmitoyl transferase 1A (CPT1A), histone acetyltransferase 1 (HAT1), lysine acetyltransferase 2A (KAT2A) and the HAT p300/cyclic adenosine monophosphate response element (CREB) binding protein (CBP) that might be especially important in the context of non-mitochondrial succinylation reactions [27–30]. For CPT1A, an enzyme localised at the outer membrane of mitochondria, it was already shown in the 1980ies that succinyl-CoA can bind to and inhibit its canonical CPT activity [31]. More recent data showed that these two enzymatic functions are regulated independently and it can be assumed that substrate availability levels direct CPT1A towards its lysine succinylation transferase activity or its canonical palmitoyltransferase activity [28]. Interestingly, CPT1A is strongly expressed in the liver, a metabolically highly active organ with high levels of lysine succinylation supporting the idea that CPT1A is a physiologically relevant succinyltransferase. For KAT2A it is suggested that the switch towards histone succinylation occurs as a consequence of a nuclear translocation of the alpha-ketoglutarate dehydrogenase (α-KGDH) complex. The nuclear presence of α-KGDH gives KAT2A access to succinyl-CoA that is produced through the enzymatic action of the α-KGDH complex. The signal that triggers nuclear entry is to our knowledge unclear for mammalian cells, but it was recently found to be regulated by light in plants [32]. It is possible though that the KAT2A succinyltransferase activity is physiologically less important than non-enzymatic succinylation as Anmangandla et al. [33] pointed out that the succinyltransferase activity of KAT2A is low compared to its acetyltransferase activity. Interestingly, using a pan-cancer dataset to study the expression of the succinylation regulators CPT1A, KAT2A, sirtuin 5 (SIRT 5), and SIRT7 showed that especially KAT2A was up-regulated in all types of tumours compared to healthy controls [34]. This indicates that KAT2A might be more important in cancer cells than under physiological conditions. Also, HAT1 is found to be elevated in several cancers and consequently, HAT1-mediated succinylation was found to be involved in tumour progression [27]. In the same study, it was shown that the presence of succinyl-CoA was able to inhibit HAT1-mediated histone H3 acetylation. Therefore, the increase in succinylation might be a consequence of the increased levels of succinate in the cancer environment. For the succinyl transferase activity of p300, conflicting reports exist [30, 35]. While one group identified the p300/CBP as lysine succinyl transferase through a double-knockout (KO) of these histone acetyl transferases, this could not be corroborated in a later study. However, Zorro Shahidian et al. [30] also noted that acetyl CoA is the preferred target of p300/CBP, and suggested that a locally increased availability of succinyl-CoA would presumably be needed to switch p300 from acetyl to succinyltransferase. The p300/CBP complex was described to have a succinylase activity specific for histone H3, which was found to destabilise in a way that transcriptional activity is enhanced [30]. In the nucleus, hyper- and hypo-succinylated peaks can generally be mapped to promoter regions and the modification of histones by succinylation and succinylated nucleosomes was found to have potent transcription-activating activities in yeast [36]. There are controversial reports regarding the question whether succinylation sites are distinct from acetylation sites (C-terminal *versus* N-terminal histone localisation), which would argue for distinct roles in the epigenetic regulation of gene expression [24, 36]. Whether lysine sites are shared between acetylation and succinylation may depend on the tissue and can be comparatively low as observed for mouse embryonic fibroblasts (MEFs) or HeLa cells (10%), or high as seen in liver mitochondria (approximately 40%) discussed in [24].

## SIRT5 and SIRT7 act as desuccinylases

Protein succinylation is a reversible PTM and the SIRT family of NAD^+^-dependent deacetylases SIRT5 was found to be an efficient desuccinylase (Figure 2). SIRTs are evolutionary conserved, however, their localisation and substrate specificity vary. In mammals, only SIRT1 and SIRT3 show robust deacetylase activity [37, 38]. SIRT5 is mainly localised in the mitochondria and was found to act as a desuccinylase and demalonylase for mitochondrial proteins [39]. Indeed, SIRT5 catalytic activities for succinylated or malonylated lysine residues are up to around 1,000-fold higher than for acetylated residues [39]. In an initial proteomic study, it was shown that SIRT5 regulated lysine desuccinylation of various metabolic enzymes in the mitochondria, but also extramitochondrial proteins like histones and ribosomes [40]. Apart from SIRT5, only SIRT7 seems to be able to desuccinylate proteins with a specificity for nuclear proteins [41–43].

SIRT5 KO mouse models display a pattern of global protein hyper-succinylation, however, initially no drastic phenotype or obvious metabolic defect was found except slightly higher serum levels of ammonia [44]. The frequency of protein succinylation greatly varies across tissues and predominately occurs in organs with high metabolic activity, such as the liver, heart, kidney, skeletal muscle, and adipose tissue [24]. Lysine succinylation of key metabolic proteins for OxPhos is evolutionarily conserved from bacteria to mammalian cells and can occur even in plant species, which suggests the functional importance of succinylation for optimal energy production and biological processes in diverse cells [45–47]. As the SIRT5 KO had been initially characterised under basal conditions the possibility existed that more challenging conditions might change the scenario [37]. Indeed, more recent findings on the pathophysiological roles of SIRT5 emphasised that SIRT5 deficiency aggravates symptoms in metabolic diseases and in tissues with high metabolic activity and SIRT5 was found to be especially important for brain, liver, heart, and brown adipose tissue (BAT) functions [48, 49].

Recent studies have intensively employed MS-based proteomic analysis combined with immunoaffinity enrichment strategies to identify potential substrates of SIRT5 and the specific succinyl-lysine residues on target proteins [50, 51]. Nonetheless, the role of site-specific effects per proteins and their regulation by SIRT5 remains unclear in most cases. Cumulative evidence confirms that nearly all TCA cycle and other metabolic enzymes are found to be succinylated with multiple succinyl-lysine sites [40] (Table 1). In general, high SIRT5 levels are associated with low protein succinylation, a preference for OxPhos and low proliferative activity, whereas the SIRT5 KO causes hyper-succinylation with often decreased enzyme activity and enhanced glycolytic activity. Thus, hyper-succinylation of mitochondrial enzymes due to loss of SIRT5 is closely linked to metabolic abnormalities in pathological and inflammatory conditions, suggesting that SIRT5 activation or overexpression could also be a tool to protect cells from metabolic stress and ameliorate the symptoms. Indeed, several studies have shown that the anti-oxidant resveratrol, which can act as a SIRT5 activator [52], as well as SIRT5 overexpression, reversed the level of succinylation, alleviated metabolic failures and prevented cell death [53–56].

**Table 1:** Targets of succinylation in the mitochondria

**Protein**	**Sites**	**Pathway**	**Model**	**Reference**
Carbamoyl phosphate synthase 1 (CPS1)	-	Urea cycle	Mouse liver	[45]
Ornithine transcarboxylase (OTC)	K88			
SDH subunit A (SDHA)	K182	TCA cycle	SIRT5 KO mouse adipose-derived mesenchymal stem cells (ADMSCs)	[57]
2-Oxoglutarate dehydrogenase E1 (OGDH)	K276			
Malate dehydrogenase 2 (MDH2)	K239, K307, K335			
Citrate synthase (CS)	-		SIRT5 KO mouse brain after subarachnoid haemorrhage (SAH)	[54]
ATP synthase	-			
Uncoupling protein-1 (UCP-1)	K56, K73, K151, K67	Thermogenesis Glutamine metabolism	SIRT5 KO mouse BAT	[58]
Glutamate dehydrogenase 1 (GLUD1)	K90, K480			
SDHA	K179, K485			
SDHB	K169			
Mitochondrial trifunctional enzyme subunit alpha (ECHA)	K351	Fatty acid oxidation	SIRT5 KO mouse heart	[50]
SDHA	K179 K335	Mitochondrial respiration	SIRT5 KO mouse heart after TAC	[59]
Hydroxyaryl-CoA dehydrogenase subunit A (HDHA)	K351			
Very long-chain acyl-CoA dehydrogenase (VLCAD)	K299, K482, K492, K507	Fatty acid oxidation	VLCAD KO and SIRT5 KO mouse	[60]
SDHA	K547	Mitochondrial respiration	Human renal cell carcinoma	[61]
IDH2	-	Reduced form of NAD phosphate (NADPH) metabolism	SIRT5 KO mouse breast cancer cells	[62]
Glutaminase (GLS)	K311	Glutamine metabolism	Human pancreatic cancer cells	[63]
	K164		Human breast and lung cancer cells	[64]
Serine hydroxymethyltransferase-2 (SHMT2)	K280	Anti-oxidant mechanism	Human colorectal carcinoma	[65]
Mitochondrial antiviral signalling protein (MAVS)	K7	Interferon response	SIRT5 KO mouse MEFs Human Embryonic Kidney 293 (HEK293T)	[66]

-: no data

## Pathophysiological consequences of mitochondrial protein succinylation

**Figure 3:**
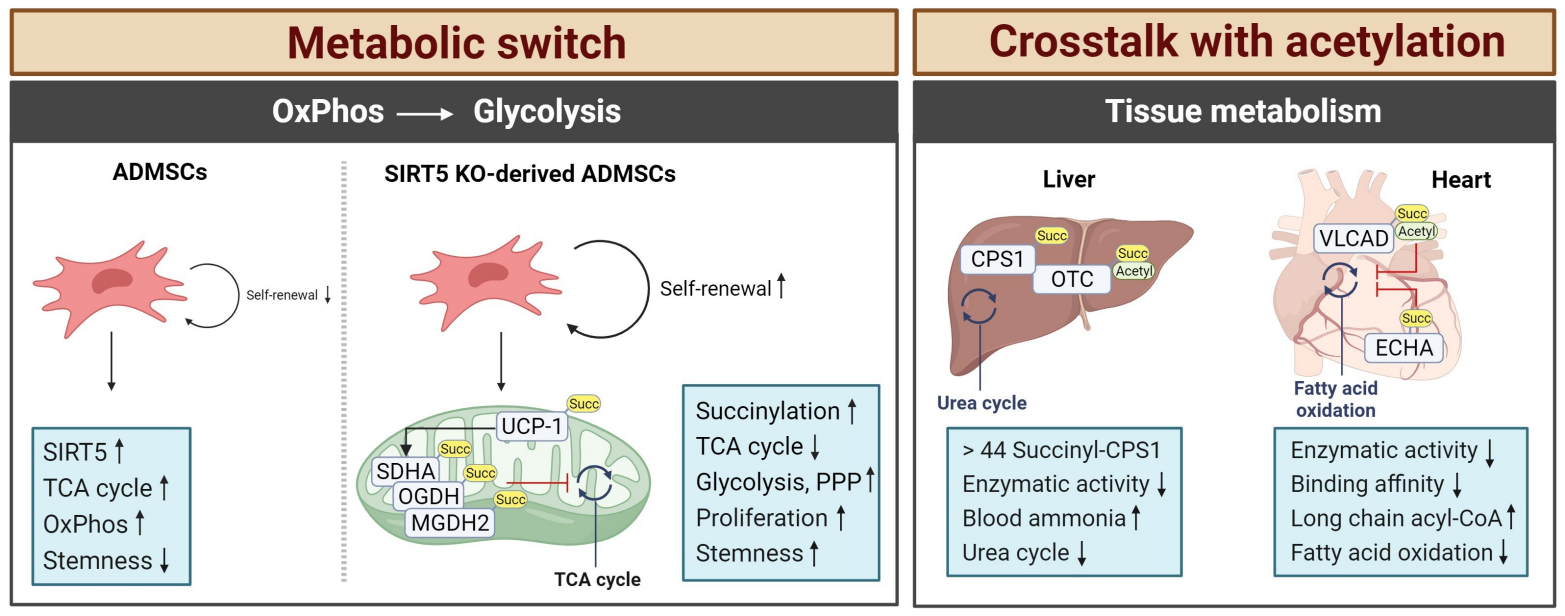
Characteristics of protein succinylation. Succinate modifications are predominantly found in tissues with high metabolic activity, such as the liver, heart, kidney, skeletal muscle, and adipose tissue. In SIRT5-deficient cells, a switch from the TCA cycle to glycolysis occurs caused by the hypersuccinylation of mitochondrial metabolic enzymes. This enhances the stemness of the cells. In tissues like brain or liver of SIRT5-deficient mice, the enzymatic activity of TCA cycle enzymes is downregulated by hypersuccinylation causing metabolic pathologies in stress situations like diet or hypertrophic cardiomyopathy and impaired cardiac functions in aging animals. Arrows depict increases or decreases in activity. Succ: succinate; MGDH2: malate dehydrogenase 2; PPP: pentose phosphate pathway. This figure was created by Biorender.com

**Figure 4:**
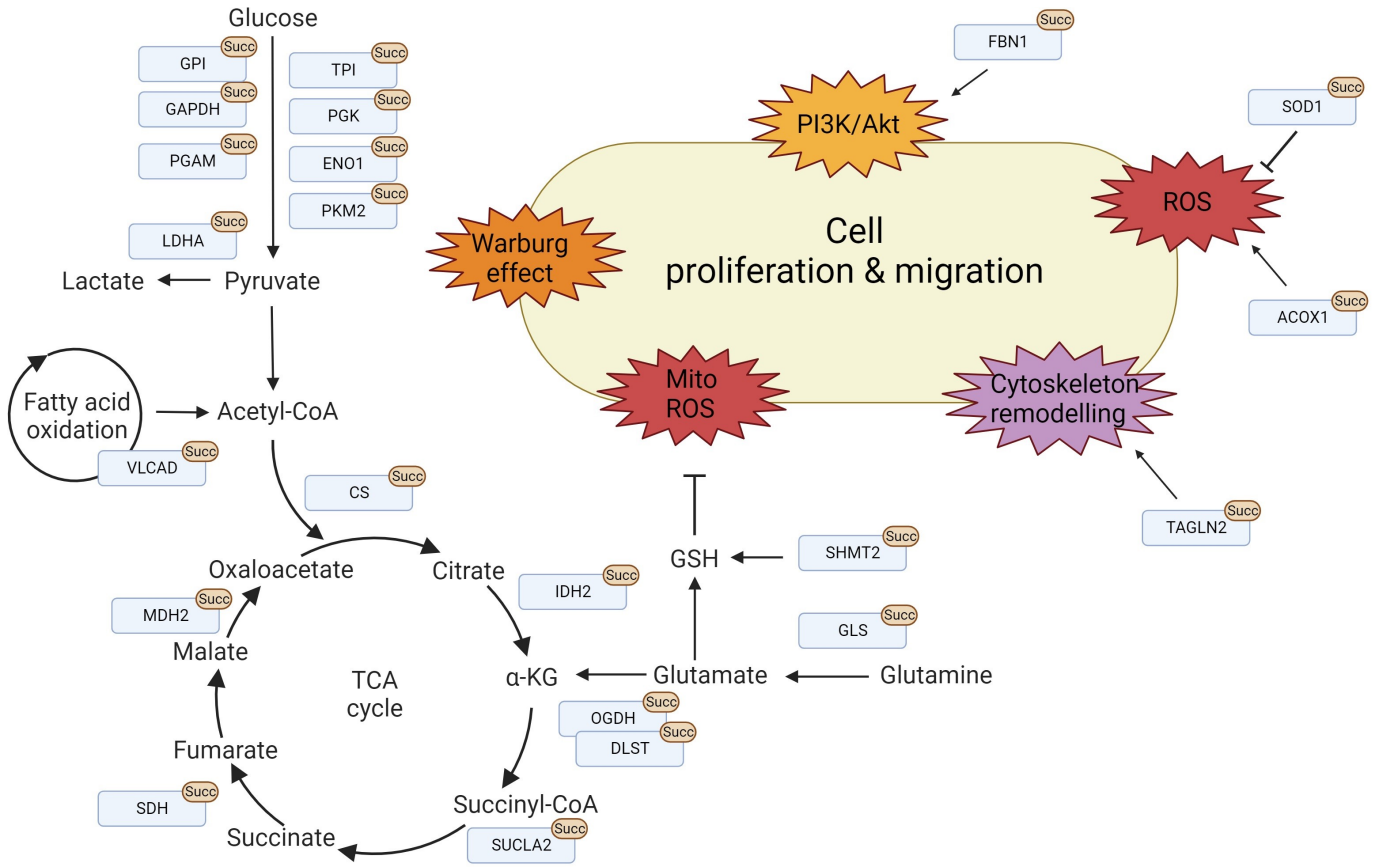
Protein succinylation promotes cell proliferation and migration. Overview of lysine succinylations found for proteins found in the mitochondria and the cytosol, respectively. These processes are mainly involved in promoting cell proliferation and migration via metabolic reprogramming, signalling activation, cytoskeleton remodelling, and ROS regulation. LDHA: lactate dehydrogenase A; TPI: triosephosphate isomerase; PGK: phosphoglycerate kinase; ENO1: enolase-1; PKM2: pyruvate kinase M2; FBN1: fibrillin 1; SOD1: superoxide dismutase 1; ACOX1: acyl-CoA oxidase 1; TAGLN2: transgelin-2; GSH: glutathione; ROS: reactive oxygen species; Akt: protein kinase B; PI3K: phosphatidylinositol-3 kinase; GPI: glucose-6-phosphate isomerase; PGAM: phosphoglycerate mutase; α-KG: alpha-ketoglutarate. This figure was created by Biorender.com

The negative net charge of the succinyl-lysine on proteins enhances the functional diversity of target proteins through conformational changes that may alter protein-protein interactions, catalytic activities, cellular signalling, and cellular localisation. In the mitochondria, enzymes involved in the central metabolic pathways are major targets of succinylation (Figure 3). These include CS, dihydrolipoyllysine-residue succinyl transferase (DLST), a component of the 2-oxoglutarate dehydrogenase complex (OGDC), OGDH, succinate-CoA ligase adenosine diphosphate (ADP)-forming subunit beta (SUCLA2), SDHA, MDH2, and IDH2. For some of these targets, the effect of the respective lysine succinylation could be linked to a pathophysiological defect (Figure 4). The crosstalk between different forms of acylation like acetylation and succinylation has been characterised for the human VLCAD, a key enzyme for fatty acid oxidation. The binding of VLCAD to cardiolipin supports the assembly of respiratory chain super-complexes on the inner mitochondrial membrane [60]. VLCAD harbours multiple lysine sites that are co-targeted by the desuccinylase SIRT5 and the deacetylase SIRT3, respectively [60]. The study revealed that both acetylation and succinylation of VLCAD at K299 within the active site negatively affected VLCAD function and flavin adenine dinucleotide (FAD) binding [60]. Moreover, succinylation of the residues K482, K492, and K507 or acetylation of K507 at the C-terminus de-localised VLCAD from the mitochondrial membrane and interfered with its binding to cardiolipin. Incubation with recombinant SIRT3 recovered the membrane binding of VLCAD and the addition of SIRT5 made the rescue more efficient. However, VLCAD catalytic activity (K299) could only be rescued by SIRT3, whereas SIRT5 did not play a role. This implies that the crosstalk between PTMs can be affected by the metabolic flux and the resulting availability of succinyl-CoA and acetyl CoA, respectively.

Mesenchymal stem cells (MSCs) are considered potential therapeutics for the treatment of ischemic diseases, where they could promote tissue repair and have beneficial immune-modulatory functions [57]. However, under normal culture conditions MSCs lose their stemness, which is caused by an upregulation of SIRT5, decreased protein succinylation, and a subsequent switch from glycolysis to OxPhos. ADMSCs from SIRT5 KO mice were found to have hyper-succinylated SDHA, OGDH, and MDH2 leading to decreased TCA cycle enzymatic activities [57]. This resulted in an increase in protein succinylation and a redirection from OxPhos to glycolysis and the pentose phosphate pathway, which supported ADMSC proliferation and survival. Therefore, local injection of ADMSCs from SIRT5-deficient mice showed enhanced therapeutic potential with increased blood flow recovery and angiogenesis in a hind limb ischemia mouse model suggesting that such genetically modified tissue could help to overcome the limitations of the *in vitro* culture conditions [57].

Although the initial SIRT5 KO characterisation did not notice a defect in thermogenesis, this could be observed when SIRT5 KO mice were kept in the cold [44, 67]. This cold intolerance was caused by a decrease in the browning capacity of their BAT, although an overall increase in adipose tissue compared to wild-type (WT) mice was noted [67]. The thermogenic capacity of adipocytes primarily relies on the activity of the UCP-1 which acts as a proton channel in the inner mitochondrial membrane and generates heat instead of ATP [68]. The thermogenic marker UCP-1 as well as the messenger RNA (mRNA) levels for proteins related to mitochondrial metabolism and biogenesis were markedly down-regulated in the adipose tissue of SIRT5 KO mice [58]. Again, hyper-succinylation of BAT proteins triggered by SIRT5 deficiency showed inhibitory effects on mitochondrial metabolism with decreased oxygen consumption rates and cell mitophagy [58]. When exposed to the cold, additional succinyl-lysine sites (K56, K73, K151, and K67) were detected and a mutational analysis confirmed their regulatory function in activity and stability. The SIRT5 KO BAT also displayed an impaired activity of GLUD1 and SDH through succinylation reflecting the broken metabolite profile with decreased glutamate oxidation and reduced levels of fumarate.

The highest amount of acyl-CoA can be detected in the heart and liver of WT mice, where it can act as a succinyl donor [50]. Indeed, SIRT5 KO mice accumulated succinylated proteins in the heart and upon aging, these SIRT5-deficient mice developed hypertrophic cardiomyopathy and impaired cardiac functions [50]. Here, a central role for the pathophysiological outcome was related to the regulation of the ECHA that is closely involved in mitochondrial fatty acid β-oxidation. An extensive succinylation of ECHA was found in the SIRT5 KO heart tissue [50]. Chemical succinylation combined with a site-directed mutation confirmed decreased catalytic activity when ECHA was succinylated at K351 close to the binding site for fatty-acyl-CoA, which consequently disturbed optimal fatty acid oxidation and accumulated long-chain acyl-CoA in the heart. Hershberger et al. [69] applied the surgical model of transverse aortic constriction (TAC) to induce cardiac hypertrophy and heart failure in SIRT5 KO mice. Consistent with the previous findings [50], the SIRT5 KO TAC model accelerated cardiac failure with high mortality and showed metabolic dysfunction including defective OxPhos, decreased fatty acid oxidation, and a low NAD^+^/NADH ratio but increased glycolysis [69]. In a second study, they further characterised the cardiac succinylome using a tamoxifen-inducible and cardiomyocyte-specific model of postnatal SIRT5 ablation and compared it to the SIRT5 whole-body KO model [59]. Succinylation continuously increased over time, showing a maximal intensity of protein succinylation at 31 weeks. Even though there was an apparent difference in the intensity of succinylation between age groups, no metabolic changes were found under either basal conditions or TAC-induced cardiac stress in this model, which was a clear difference from the previously used whole-body KO. This suggests that cooperative effects between organs like the heart and kidney need to be considered when interpreting pathophysiological results using whole-body KO mice models. Interestingly, they noted that only three lysine residues K179 and K335 on SDHA and hydroxyaryl-CoA dehydrogenase subunit A (HDHA) K351, were identified as SIRT5 substrates between their postnatal heart-specific SIRT5 KO and a germline deletion of global SIRT5 [59].

SIRT5-mediated succinylation is a critical regulator of metabolic stress in acute brain injury after SAH. Both, patients with SAH and endovascular perforation-induced SAH mice markedly increased lysine succinylation in the brain cortex due to the decreased expression of SIRT5 [54]. Here, the metabolic enzymes CS and 4 subunits of ATP synthase of the complex V in the electron transport chain (ATP2a2, MP68, TAF3, and ATP5F1e) were identified as the substrates of SIRT5. Their succinylation resulted in the deactivation of the enzyme resulting in decreased ATP production, enhanced ROS, and a drop in brain tissue pH level. This eventually resulted in neuronal cell death with severe neurological defects. In the liver, the majority of succinylated proteins are associated with the TCA cycle, fatty acid oxidation, and the urea cycle [70]. An initiator of the urea cycle producing carbamoyl phosphate from ammonia, CPS1, was measured to contain as many as 44 succinyl-lysine sites [45]. An early study had shown that succinylation on CPS1 reduced its enzymatic activity in SIRT5 KO mice compared to the WT control [39]. Additionally, SIRT5 KO mice failed to clear ammonia from the blood during fasting due to the defective CPS1 activity [71]. OTC involved in the second step of the urea cycle was also found to be succinylated at K88 [45], a lysine previously known to be a target of acetylation [72] reducing the substrate binding and consequent inhibition of OTC activity [73]. Recent studies suggested that loss of SIRT5 raises the risk of obesity-related metabolic dysregulation with excessive lipid accumulation in the liver [74]. Using a C_12_-rich diet, SIRT5 KO mice developed periportal macrovascular steatosis with defective mitochondrial C_12_ fatty acid oxidation. Consequently, liver-specific overexpression of SIRT5 in a type 2 diabetes obesity mouse attenuated hepatic steatosis and reduced the lipid contents of hepatocytes due to improved fatty acid oxidation and glycolytic flux caused by decreased protein succinylation [55]. Interestingly, the authors observed a clear difference between SIRT5-mediated malonylation and succinylation. While the glycolysis/gluconeogenesis pathway was modified through protein malonylation, protein succinylation was primarily found for protein targets associated with the OxPhos pathway [55].

## Cancer-related mitochondrial protein succinylation

Accumulated succinate has been regarded as a prognostic marker for various diseases with metabolic dysfunction such as rheumatoid arthritis, diabetes, atherosclerosis, or cancer [75]. Most importantly, studies have documented the oncogenic capacity of the succinylation of specific proteins, as succinylation of TCA cycle enzymes often supports the metabolic shift towards glycolysis [76]. As a result of the increased metabolic rate, tumours exhibit high levels of endogenous ROS [77]. Recently it has been suggested that tumour cells exploit SIRT5-mediated regulatory mechanisms as tumour inducer, however, the expression and effect of SIRT5 is highly varied depending on the tissue context [76]. Tumour cells can for example promote an antioxidant response through enhanced SIRT5-mediated protein desuccinylation to avoid ROS-induced apoptosis [62]. Increased levels of SIRT5 expression were for example detected in human breast cancer samples, and the resulting IDH2 desuccinylation accelerated its enzymatic activity and produced increased amounts of NADPH, thus combating the oxidative stress [62, 78]. This was corroborated by the pharmacological inhibition of SIRT5, which significantly decreased tumour size in mouse xenograft models of breast cancer [62]. Also, in malignant gliomas, mutations in IDH1/2 cause an excessive accumulation of the oncogenic metabolite R-2-hydroxyglutarate (R-2HG) [79]. Because R-2HG is a structural analogue for succinate, it can bind to SDH and interfere with SDH activity thus promoting succinate accumulation and hyper-succinylation [80]. Due to the SDH inactivation, IDH1/2 mutation-bearing gliomas show impaired respiration and recruit B-cell lymphoma 2 (Bcl-2) to the mitochondrial membrane to overcome cellular apoptosis. Consistent with this, SDHA inactivated by a K547 desuccinylation was found to promote the development of renal carcinoma [61]. This was corroborated by introducing a K547R mutation, which abolished the interaction with SDHA and prevented SDH activity.

Antioxidant effector molecules in cancers are therefore getting recognised as potential targets of protein succinylation. Mitochondrial glutaminolysis for example is crucial for cancer metabolism to maintain redox balance by generating the key antioxidant GSH to protect against oxidative injury and anabolic utilisation [81]. Recently, Tong et al. [63] reported the specific function of lysine succinylation in the kidney-type GLS which is highly present in human pancreatic ductal adenocarcinoma (PDAC). GLS K311 is required to form catalytically active GLS oligomers therefore supporting PDAC development in mice. Mechanistically, p38 mitogen-activated protein kinase (MAPK) kinase, activated by hydrogen peroxide (H_2_O_2_), serine-phosphorylates SUCLA2 leading to the dissociation of SUCLA2 from GLS. This consequently promoted GLS K311 succinylation and the generation of NADPH and GSH in AsPC-1 cells [63]. In human breast and lung cancer cell lines, SIRT5-mediated GLS desuccinylation at K164 was found to stabilise GLS and prevent ubiquitin-mediated protein degradation so that high levels of GSH could be produced [64]. SHMT2 is a core member of the serine catabolism required for one-carbon units to produce DNA/RNA as well as GSH to reduce intracellular ROS [82]. Increased SHMT2 expression is therefore associated with cancer [82]. Desuccinylation of K280 on SHMT2 by SIRT5 was found to be essential to form the active tetramer and supported colorectal tumour progression due to sustained levels of GSH [65]. Similarly, a human colorectal carcinoma showed reduced SHMT2 succinylation under metabolic challenges with serine/glycine deprivation and a mutational approach confirmed that SHMT2 K280E abolished the binding affinity of pyridoxal phosphate (PLP), a critical cofactor to activate the serine/glycine pathway.

Since protein succinylation and its regulation by SIRT5 were discovered, this PTM has been recognised for its central role in regulating metabolism in response to environmental changes. Dysregulation of succinylation significantly impacts the targeted enzymatic activities and worsens physiological symptoms in disease models such as cancer, obesity, and cardiac failure. These observations opened up the possibility of using protein succinylation as a diagnostic marker or potential therapeutic target for a broad range of pathologies.

## Protein succinylation in the cytoplasm

In contrast to the mitochondria where succinate concentrations can be high, many cytoplasmic protein succinylations are mediated by the succinyltransferases CPT1A and HAT1 [27, 28]. In 2018, Kurmi et al. [28] first identified CPT1A-catalysed succinylation in 293T cells overexpressing CPT1A and showed that the lysine succinyl transferase activity is independent of its CPT activity. Nearly half of the 247 identified proteins were located in the cytosol highlighting that protein succinylation is not mitochondria-specific [28]. Similar to mitochondrial protein succinylation, succinylation of cytoplasmic proteins frequently targets metabolic enzymes and often enhances their glycolytic activity (Figure 3, Table 2). Thus, succinylation supports tumorigenesis by facilitating the shift towards glycolysis. When HAT1 was first characterised as a succinyltransferase, it was also found to drive tumorigenesis [27]. Most of its non-histone succinylated targets are involved in glycolysis such as GPI, TPI, glyceraldehyde 3-phosphate dehydrogenase (GAPDH), PGK, PGAM, ENO, and pyruvate kinase M (PKM). Consequently, a knock-out of HAT1 in HepG2 cells significantly decreased glycolytic activity and resulted in decreased glucose consumption and production of lactate and glycolytic by-products such as pyruvate, ATP, and NADH [27]. Other major targets of cytoplasmic protein succinylation are a variety of proteins responsible for maintaining the cytoskeleton, cell integrity, and migration. Because many of these proteins play a role in migration and metastasis, the attachment of succinylation might alter protein function and facilitate tumour progression.

**Table 2:** Targets of cytoplasmic succinylation

**Protein**	**Sites**	**Pathway**	**Model**	**Reference**
SOD1	K123	Anti-oxidant mechanism	Lung tumour cells HEK293T	[83]
ACOX1	K89, K437, K488, K500, K537	Fatty acid oxidation	SIRT5 KO mouse Human hepatocellular carcinoma (HCC) cancer samples	[84]
PKM2	K433	Glycolysis	Human cancer cells Colon cancer specimens	[85]
	K311	PKM2-HIF-1α-IL-1β	Bone marrow-derived macrophages (BMDMs)	[86]
	K498	Anti-oxidant mechanism	Human lung carcinoma cells	[87]
ENO1	K80, K81 K355	Cell proliferation	Breast cancer cells	[28]
PGAM1	K99	Glycolysis	Human pancreatic cancer cells	[27, 88]
LDHA	K118		Prostate cancer cells	[89]
	K222		Human gastric cancer (GC) samples	[90]
TAGLN2	K40	Cytoskeletal rearrangement	Human glioblastoma samples and cells	[91]
FBN1	K672	Transforming growth factor β1 (TGFβ1)-PI3K/Akt	Huma primary gastric adenocarcinoma samples	[92]
S100A10	K47	Tumour migration and invasion	Human GC tissues	[93]

## Cancer-related changes in protein succinylation

Succinate participates in several metabolic pathways and is involved in maintaining ROS homeostasis [94]. Therefore, the variation of lysine succinylation levels reflects both, cellular metabolic activity and redox status and conversely protein succinylation can modulate cellular redox conditions. In tumorigenesis, succinylation promotes ROS production by regulating the activity of redox enzymes in the mitochondria but also in the cytoplasm. SOD1 is central to this process as it can deactivate the superoxide radical anion into hydrogen peroxide before further reduction with catalase [95]. In a study using SIRT5 KO mice, SOD1 was found to be a target of succinylation [83]. Further investigations showed that SOD1 desuccinylation is mediated by direct binding of SIRT5 to SOD1 and that succinylation decreases its activity, causing enhanced ROS levels [83]. Interestingly, it was found in this study that the mutation of the SOD1 succinylation site inhibited the growth of the lung tumour cell line H1299 and that H1299 cells overexpressing succinylated wildtype SOD1 had a growth advantage.

Peroxisomal ACOX1 transfers electrons from 1,5-dihydroflavin adenine dinucleotide (FADH_2_) to molecular oxygen which results in the production of H_2_O_2_ through the denaturation of very-long-chain acyl-CoAs to 2-trans-enoyl-CoAs. Crystal structure analysis indicated that ACOX1 dimerisation is required for its catalytic activity [96]. Succinylation of ACOX1 at several lysine residues had been found to promote its enzymatic activity and facilitate dimer formation while SIRT5-mediated desuccinylation inhibited the formation of the active homodimer [84]. As a result, a SIRT5 knock-down caused an accumulation of H_2_O_2_ and enhanced oxidative damage due to the activation of ACOX1. High peroxisomal activity is found especially in the liver where it contributes to fatty acid degradation. To corroborate the relevance of ACOX1 succinylation clinically, HCC samples were investigated [84]. Indeed, in HCC samples SIRT5 levels were significantly reduced, which resulted in enhanced succinylation of ACOX1 and increased ACOX1 activity. The increased levels of oxidative DNA damage resulted in poor HCC survival and higher SIRT5 expression was connected to a more favourable prognosis.

Among the glycolytic enzymes, PKM2 is a rate-limiting glycolytic enzyme that plays a central role in regulating cancer metabolism and tumour growth by mediating the conversion of phosphoenolpyruvate (PEP) into pyruvate among other functions. Qi et al. [85] investigated the role of lysine succinylation for mitochondrial translocation under conditions of glucose starvation and cellular stress. Succinylation of PKM2 enhanced its binding to the VDAC3 and prevented VDAC3 degradation. VDAC3 stabilisation resulted in an increase in mitochondrial permeability, which promoted ATP export to the cytosol under conditions of nutritional depletion. Interestingly, cells from colon cancer patients showed a comparable correlation between VDAC3 levels and PKM2 activity [85]. Pharmacological inhibition of PKM2 mitochondrial translocation was also able to inhibit tumour development, highlighting the central role of PKM2 succinylation.

Overexpression of the succinylase CPT1A identified the glycolytic enzyme ENO1 as the most heavily succinylated protein [28]. Using the proliferation of the BT474 breast cancer cell line as a read-out, it was shown that CPT1A-mediated succinylation inhibited the catalytic activity of ENO1, but promoted glutamine-independent cell proliferation, a rescue mechanism of ENO-deficient MDA-MB231 breast cancer cells that had previously been reported [28, 97]. PGAM1 is a glycolytic enzyme that catalyses the conversion of 3-phosphoglycerate to 2-phosphoglycerate [98]. It is abundantly expressed in various types of cancer and is considered a key factor in cancer metabolism [98, 99]. A knock-out of HAT1 in HepG2 cells significantly decreased the glycolytic activity resulting in decreased glucose consumption, production of lactate, and the generation of glycolytic by-products such as pyruvate, ATP, and NADH [27]. In the absence of HAT1, PGAM1 succinylation and its activity were reduced in tumour cells and re-expression of a mutant that was not permissive to lysine succinylation (PGAM1-K99R) in PGAM1-KO cells, therefore showed reduced tumour cell proliferation [27]. Aspirin is considered to have a preventive function in cancer [100]. More recently, Wang et al. [88] linked the chemo-preventive properties of aspirin in HCC cells to the succinylation of PGAM1. Treatment with aspirin caused a nuclear factor κB (NF-κB)-mediated decrease in the expression of HAT1, which subsequently resulted in impaired activity of PGAM1. Consequently, PGAM1 succinylation was reduced and the decreased level of glycolysis ultimately inhibited cancer progression [88]. This shows that already existing drugs may have the ability to change protein succinylation, a fact that should be further investigated.

LDHA is a central step of cellular glycolysis and catalyses the conversion of pyruvate into lactate. High expression of LDHA is correlated with tumour progression in several cancer types, like renal cancer, pancreatic cancer, breast cancer, or GC [101]. LDHA has been identified as a substrate for SIRT5-mediated desuccinylation. Decreased expression of SIRT5 and consequently enhanced levels of LDHA succinylation on LDHA-K118, were found to negatively correlate with the survival rates of patients as the hyper-succinylation enhanced its activity and exacerbated prostate tumour proliferation and migration [89]. A global succinylome study using the prostate cancer cell line PC-3M, identified 7 succinylated lysine sites for LDHA. Mutational analysis, however, showed that only K118 was important for LDHA activity and increased its activity when succinylated [89]. Overexpression of CPT1A in the GC cell line AGS was found to hypersuccinylate LDHA on K222 [90]. However, this modulated LDHA stability rather than its activity. Since the residue K222 is exposed in the protein structure, succinylation of this residue seems to interfere with the interaction between LDHA and the ubiquitin-binding protein p62 thereby inhibiting LDHA degradation. Stabilisation of LDHA resulted in enhanced glycolysis and promoted tumour cell progression, metastasis, and growth [90].

Apart from the well-described influence of lysine succinylation on metabolic enzymes, protein-protein interactions can be modified by the attachment of a succinate moiety to a protein. TAGLN2 for example is responsible for regulating the cytoskeleton of immune cells and endothelial cells and stabilising the actin cytoskeleton which supports for example the formation of an immune synapse [102]. However, upregulation of TAGLN2 is also found in many cancer types and is correlated with clinical stage, neural invasion, and tumour size (reviewed in [102]). In glioma tissue, hypersuccinylation of TAGLN2 seemed to play a critical role in promoting migration and angiogenesis. Using the glioma endothelial cell line U87 it was found that succinylated TAGLN2 recruits and represses the function of thymosin beta 4 X-linked, an inhibitor of actin polymerisation, to initiate cytoskeletal remodelling and glioma migration [91]. These data suggest that the succinylation status of TAGLN2 can serve as a diagnostic biomarker or even a therapeutic target in the future. Despite the important function of TAGLN2 for immune cells, the function of its succinylation has not been investigated yet.

FBN1 is a large calcium-binding glycoprotein that serves as a structural component of microfibrils in the extracellular matrix [103]. FBN1 is highly expressed in GC patients and the succinylation of FBN1 on K672 and K799 was found to be upregulated in GC patients compared to normal tissue controls [92]. The matrix metalloproteinase 2 (MMP2) can degrade FBN1 [104], but succinylation of FBN1 on K672 disrupts this interaction with MMP2 in the tumour microenvironment causing an accumulation of FBN1 [92]. This accumulated FBN1 then interacts with the integrins β3 and β5, triggering the release of TGFβ1 and the activation of the PI3K/Akt pathway, which ultimately facilitates cell proliferation and tumour progression [92]. Another interesting example is the modification of the S100 calcium-binding protein A10 (S100A10). S100A10 plays an important role in maintaining calcium homeostasis, apoptosis, motility, cytoskeleton interaction, angiogenesis, and inflammation [105, 106]. S100A10 is also an important negative regulator of Toll-like receptor 4 (TLR4) signalling as S100A10 deficient mice are more susceptible to lipopolysaccharide (LPS)-induced lethal shock [107]. In addition, the overexpression of S100A10 is related to tumour progression [108], and the quantitative polymerase chain reaction (PCR) confirmed that S100A10 is associated with the malignant transformation of kidney cells. In a recent study, Wang et al. [93] found that S100A10 expression was up-regulated in tissues from GC patients and increased levels of succinylated K47 could be identified in GC tissues by MS. They further validated that CPT1A and SIRT5 catalyse succinylation and desuccinylation of S100A10-K47. Succinylated S100A10 was protected from ubiquitylation-mediated proteasomal degradation. As a consequence, accumulated S100A10 promoted cancer cell invasion and migration. This was corroborated by overexpressing K47E or K47R mutations in the GC cell line MGC-803, where an increase in cell migration was observed compared to cells expressing WT S100A10 [93].

The function of protein succinylation on both metabolic enzymes and non-enzymatic target proteins has not been studied much. Given the strong effects that are observed in tumour cells, this will be an exciting new field for research of cancer and immunology.

## Histone succinylation

**Figure 5:**
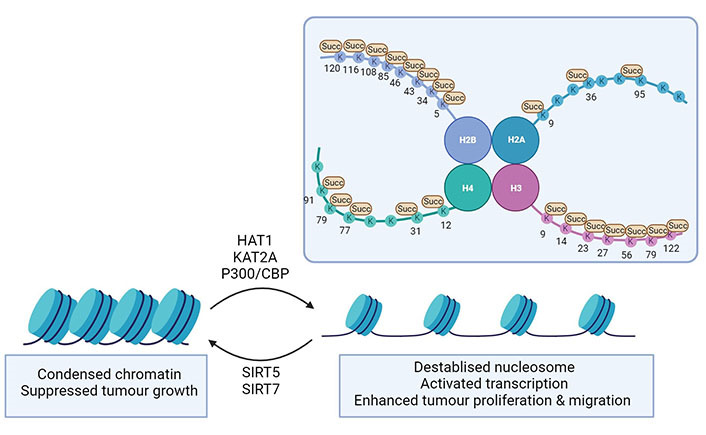
Histone succinylation in diseases. Succinylation on histone lysine sites (depicted as K) promotes chromatin remodelling, reduced nucleosome stability, and transcriptional activation. This ultimately leads to enhancing cell proliferation and migration in cancer progression or facilitating HBV transcription and replication in viral infection. Succinylases such as HAT1, KAT2A, and p300/CBP take part in the process of enzymatic succinylation of histones. The desuccinylases SIRT5 and SIRT7 can reverse succinylation and decrease chromatin accessibility. This figure was created by Biorender.com

Several histone PTMs such as acetylation, methylation, phosphorylation, and ubiquitinylation have been identified, but the importance of lysine acetylation has been best studied [3]. Each core histone domain contains highly conserved, positively charged lysine residues that can interact with negatively charged DNA. When acetylation is added, the positive charge of the lysine residue is neutralised and chromatin remodelling occurs. As lysine succinylation introduces a comparatively large structural moiety (100 Da *versus* 42 Da for acetylation) to the protein [37], a substantial change in histone packaging and stability can be expected. Indeed, histone succinylation facilitates DNA unwrapping and promotes the accessibility of transcription factors [36] (Figure 5). Succinylation on lysine residues located on the spherical core structure can directly reduce the nucleosome stability, causing chromatin relaxation, and stimulating transcription. Histone modifications have been identified for H2A, H2B, H3, and H4 and most of the identified succinylation sites are located in the C-terminus and globular domain of the respective histone [36, 109, 110]. The mitochondrial desuccinylase SIRT5 and the deacetylase SIRT7 can both desuccinylate histones, as a subcellular fractionation study found SIRT5 to be also present in the nucleus [40]. Several histone-specific sites on H2B, H3, and H4 have been identified as SIRT5 targets [111] (Table 3). As observed in the mitochondria, histone succinylation can even occur as a non-enzymatic process if succinyl-CoA is abundantly present [9]. Due to the structural and conformational dynamics of histone, some lysine residues in the globular domain or C-terminus of histones are more accessible to the succinyl-CoA [36]. By treating HeLa cells with isotopic succinate for 24 h, Xie et al. [112] were able to identify the isotopic succinylation (sodium D4-succinate) on histones. This indicates that succinate can in principle be transported from the extracellular environment into the nucleus where it could serve as a source of histone succinylation [112].

**Table 3:** Targets of succinylation in the nucleus

**Protein**	**Sites**	**Protein**	**Model**	**Reference**
H2A	K9, K36, K95	-	*In vitro* study	[112]
H2B	K34	Destabilised nucleosome	*In vitro* study and budding yeast	[113]
	K5, K43, K46, K85, K108, K116, K120	-	Review	[114]
H3	K122	Destabilised nucleosome Active transcription	Breast cancer cells	[115]
		Active transcription	Human pancreatic cancer cells	[27]
		Active hepatitis B virus (HBV) transcription	HBV-infected human liver	[41]
			HBV-infected human hepatocytes	[116]
	K79	Active transcription	Human glioblastoma cells	[29]
		Active transcription β-catenin stabilization	Pancreatic cancer cells	[117]
		Active HBV transcription	Chimeric mice, HBV-infected human hepatocytes	[118]
	K9, K14, K23, K27, K56	-	*In vitro* study	[36, 112, 114]
H4	K77	DNA unwrapping Active transcription	*In vitro* study	[109]
	K12, K31, K79, K91	-	*In vitro* study	[111, 112]

-: no data

## Histone-succinylation as a driver of tumorigenesis

Since the addition of succinylation adds a negative charge on the lysine sidechain, it is bound to cause a considerable change in the chromatin structure. Zorro Shahidian et al. [30] discovered that succinylation of H3 at K122 caused an enrichment in transcription start sites (TSS) in the human breast cancer cell line MCF7. This was corroborated by an *in vitro* transcription assay, showing that the addition of succinylation on H3 K122 alone stimulated transcription [30]. H3 K122 succinylation can be mediated by HAT1 and plays an important role in epigenetic gene regulation [27]. Knocking out HAT1 in a nude mice tumorigenicity model reduced H3 K122 succinylation and inhibited the growth of HepG2 and PANC1 cancer cells, suggesting that H3 K122 succinylation is important for tumourigenesis. Wang et al. [29] found the H3 K79 succinylation to be strongly enriched around the TSS of the glioma cell line U251. By gene set enrichment analysis, they examined the signalling pathways involved in H3 K79 succinylation in their promoter regions and found proliferation and growth-related signalling cascades resulting in the activation of PI3K and activator protein 1 (AP1). Reduced histone succinylation of H3 K79 on the other hand suppressed the growth of glioblastoma cells [29]. A similar observation was made for PDAC, where KAT2A-mediated H3 K79 succinylation was found to be enriched in the promoter region of the adaptor protein 14-3-3γ ultimately causing the stabilisation of β-catenin [117]. Under physiological conditions, β-catenin is degraded through ubiquitin-dependent proteasomal degradation, and 14-3-3γ expression stabilises β-catenin and subsequently increases the expression of its downstream target’s cyclin D1, cellular myelocytomatosis oncogene (cMYC), glucose transporter type 1 (GLUT1) and LDHA. By regulating the expression of this set of genes, cell metabolism, glycolysis, proliferation, migration, and invasion of PDAC are influenced [117].

Histone succinylation does not only play a role in tumour progression but is also associated with viral infection. Two lysine residues on H3 (K79 and K122) were found to regulate HBV transcription and replication [41, 116]. HBV is a small DNA virus that targets hepatocytes [119] and covalently closed circular DNA (cccDNA) serves as a template for HBV replication by hijacking the host’s transcriptional machinery [120]. It has been discovered that cccDNA succinylation upregulated HBV transcriptional activity [118]. KAT2A is recruited to the minichromosome and succinylates H3 K79 to facilitate HBV replication [116]. Interestingly, the cytokine interferon-alpha (IFN-α) that interferes with viral replication suppressed succinylation of H3 through regulating KAT2A activity. The KAT2A-mediated H3 K79 succinylation is also supported through an interaction between KAT2A and the HBV core protein (HBc) [118]. Likewise, Yu et al. [41] identified SIRT7 as a potential desuccinylase that can be recruited to the cccDNA site to interact with HBc and remove the H3 K122 succinylation. As a result, the structure of cccDNA changes and leads to the epigenetic silencing of cccDNA transcription and the failure of HBV replication. In summary, KAT2A and SIRT7 are able to manipulate H3K79 and H3K122 succinylation of HBV cccDNA, respectively. This implies that targeting and silencing of cccDNA via histone modifications such as succinylation might be a potential antiviral strategy for the prevention or treatment of HBV infection.

## Outlook

PTMs are generally understudied in the context of immune regulation, although pattern recognition molecules and their downstream adaptors and signalling molecules can be influenced by PTMs like phosphorylation or ubiquitination [121]. The reported changes in metabolic activity now draw our attention toward the possibility that the attachment of metabolites also takes part in the fine regulation of immune cell responses. While succinate can act as a signalling molecule by itself either through receptor binding or protein interactions, a two-fold increase in protein succinylation was observed as a consequence of LPS treatment of mouse bone marrow-derived macrophages [23]. This is not only due to the described rise in succinate levels due to the disruption of the Krebs cycle, but also due to a decrease in SIRT5 levels [23]. Proteomic analysis of the SIRT5 KO mouse revealed thousands of potential SIRT5 interaction sites, especially on metabolic enzymes, but the function of the succinylation and its importance have only been clarified for a few of them. To date, not much is known about the targets of protein succinylation during infection and it is unclear whether targets other than metabolic enzymes play an important regulatory role during infections.

The M2 isoform of PKM2 regulates the final and rate-limiting steps of glycolysis. Unexpectedly, it was found to have a central role in infections as it regulates HIF-1α activity and consequently the levels of IL-1β expression [122]. PKM2 also enhances signal transducer and activator of transcription 3 (STAT3) activation and critically controls the development of T helper 17 (TH17) cells and subsequently is found in auto-immunity [123, 124]. These so-called moonlighting functions are critically regulated by PTMs like acetylation, phosphorylation, and SUMOylation [125]. Succinylation of PKM2 at K498 was found to influence ROS, tumour growth, and cell proliferation [87] and Wang et al. [86] therefore investigated if the ROS response of inflammatory macrophages might also be influenced by PKM2 succinylation. They showed that SIRT5-mediated desuccinylation of PKM2 on K311 promoted classical PKM2 activity in its tetrameric formation and reduced enzyme dimerisation and its action as a protein kinase resulting in an increase in IL-1β production from SIRT5 KO macrophages. In a colitis model using dextran sulphate sodium (DSS) in mice, the absence of SIRT5 aggravated the diseases suggesting that the modulation of protein succinylation levels might be helpful in certain pathological contexts [86].

Viral infections are sensed by cytosolic RNA sensors of the retinoic acid-inducible receptor family [retinoic acid-inducible gene I (RIG-I) like receptors] such as *RIG-I* or melanoma differentiation-associated gene 5 (*MDA5*) [126]. Downstream of these sensors the adaptor protein MAVS is a central protein to eventually trigger an interferon response [127]. It was found that SIRT5 interacts with MAVS and desuccinylates K7. Interestingly, the presence of SIRT5 impaired the activation of the adaptor MAVS which is a central factor for a correct response of the innate immune system towards viral infections and disrupted the anti-viral response of macrophages and DCs [66]. Here, disruption of SIRT5 was shown to boost the host immune response.

Indeed, SIRT5 deficiency does not limit the overall ability of the innate immune system to respond to a bacterial infection with respect to immune cell differentiation of B cells and T cells, cytokine production, and macrophage proliferation [128]. Challenging mice with the TLR4 ligand LPS or infection of the animals with *Klebsiella pneumoniae* (non-severe pneumonia) or *E. coli* (peritonitis model) did not cause significant differences between WT and KO animals. This is important information, as the development of SIRT5 inhibitors for oncologic purposes would need to guarantee that the immune system is not compromised by the treatment.

Interestingly, a recent paper on severe acute respiratory syndrome coronavirus 2 (SARS-Cov-2)-infected cells revealed an infection-specific change of the succinylome of both host and viral proteins [129]. Again, the main target proteins in the host cell were metabolic enzymes belonging to glycolysis, TCA cycle, fatty acid oxidation, and mitochondrial ADP/ATP transport. Through a bioinformatic characterisation of the interactome, the authors found that the structural protein nonstructural protein (NSP14) of the virus, an exonuclease and RNA methyltransferase, could potentially interact with SIRT5 in the cytoplasm. This was experimentally verified by co-immunoprecipitation (co-IP). Their data suggest that the interaction suppresses SIRT5 activity as it was further found that NSP14 overexpression increased protein succinylation, while overexpression of SIRT5 on the contrary inhibited viral replication [129]. Thus, microbial organisms seem to be able to specifically change the host cell proteome of succinylated proteins to establish a niche for replication. It can be anticipated that similar mechanisms will be found in the future for bacterial pathogenicity factors. The interaction of the viral protein NSP14 of SARS-Cov-2 with SIRT5 and the subsequent induction of protein succinylation suggested that this mechanistic link might provide an interesting target and treatment option [129]. The authors investigated if inhibition of succinylation of specific proteins had a measurable influence on viral replication. CPT1A and proliferator activated receptor coactivator-1 alpha (PGC1a) can indirectly support the desuccinylation activity of SIRT5 and so does the inhibition of adenosine monophosphate-activated protein kinase (AMPK) [130]. Therefore, AMPK and CPT1A inhibitors and CPT1A and PGC1a activators were tested for potential antiviral activity. Although the inhibitory activity could also have been an indirect effect, the data are promising as they suggest that the identification of highly succinylated protein might suggest targets and the use of already existing inhibitors without the need to generate inhibitors that exert succinylation-specific actions.

Succinate has been known for a long time as a metabolite that accumulates during oxygen deprivation and is described to be a universal signature for ischemia [131]. The heart critically depends on mitochondrial activity and the supply of oxygen. Myocardial infarction (MI) causes reperfusion injury due to the production of ROS [132]. MI is a major cause of death and there are currently no therapeutical approaches to limit organ damage to prevent heart failure. During ischemia, TCA cycle metabolites accumulate suggesting that they might be a potential target. Accumulation of succinate is a driver of ROS production as it causes the reverse flow of electrons between complex I and II [reverse electron transfer (RET)] in a non-functional Krebs cycle and impaired OxPhos reaction [131, 133]. Biochemically this happens when succinate accumulates in cells that are not generating sufficient ATP through OxPhos. Under these conditions, electrons do not flow through the electron transport chain, but the electrons travel in the reverse direction at complex I resulting in ROS production. Once blood flow and tissue oxygenation are restored, the accumulated succinate results in RET and increased levels of ROS [131]. To optimise reperfusion therapy, additional measures are critically needed to protect the myocardium from further damage and it can be discussed that a better understanding of succinate activities could be of help. SIRT5 inhibitors are currently being developed for oncologic conditions but might be of potential interest for other conditions with an increase in succinate levels [128].

SIRT5 is involved in processes that are necessary for cellular homeostasis, like metabolic pathways and the regulation of ROS levels and its function is therefore important for the brain and the heart and in the response to environmental stress and cancer, SIRT5 can be either protective or cancer-promoting, depending on other environmental factors [134]. SIRT5 is therefore an attractive target to generate inhibitors as well as activators for the SIRT5 enzymatic activity [134]. Additionally, such chemical tools will provide us with a better understanding of cell-specific SIRT5 actions.

As mentioned earlier, the chemo-preventive properties of aspirin in HCC cells can be linked to an aspirin-triggered decrease in NF-κB activation that resulted in a lowered expression of HAT1. As a consequence, PGAM1 activity and cellular glycolytic activity were lowered which contributed to inhibition of cancer progression [88, 100]. These data are promising and highlight the development of drugs capable of modulating the expression of writers and erasers of PTMs can be a strategy to find novel treatment options. There are already examples where *in silico* drug screening for modulators of lysine-modifying enzymes such as ubiquitination inducers, lysine deacetylase inhibitors, and lysine demethylase inhibitors were successfully performed [135]. One of the examples is KPM-2, a SIRT2 inhibitor designed based on the enzyme catalytic mechanism of NAD-dependent deacetylase SIRT2 [135]. The inhibitory effect of KPM-2 was validated in breast cancer cell lines as it induced acetylation on α-tubulin, a substrate of SIRT2. Such an approach seems advantageous also for targeting pathologic succinylation that occurs as a consequence of HAT overexpression. Thus, similar approaches could be envisioned for KAT2A where the nuclear translocation of -KGDH is potentially a consequence of cancer and enhanced glycolysis.

Most of the mouse data that investigate that investigated the succinylation of mitochondrial proteins were generated using SIRT5 KO models to induce protein succinylation, and only a few studies have addressed the physiologically more relevant roles of elevated levels of mitochondrial succinate and the resulting functional consequences, as succinate can accumulate due to SDH or succinyl-CoA ligase (SCL) dysfunctions [56]. SCL deficiency for example is a mitochondrial DNA depletion syndrome that causes defects in the heart, liver, brain, and muscle. Patients with progressive mitochondrial encephalomyopathy due to the genetic mutations in *SUCLA2* or subunit alpha (*SUCLG1*) show a dramatic increase in protein succinylation compared to healthy controls. A *SUCLA2*-deleted zebrafish model mimicked this global hyper-succinylation observed in SCL patients and was characterised by impaired mitochondrial function with reduced oxygen consumption rate and decreased survival of larvae, which implies the pathological effects of succinylation [56]. Physiologically, however, succinate is required for the functionality of M1 macrophages, activated DCs, and T cells, cells that are important for anti-tumour activities [136]. Thus, future work will need to focus on both, the physiological role of lysine succinylation and its importance for correct cell function as well as on investigating the abnormalities of succinylation patterns in progressive diseases.

## Conclusions

Succinate is a comparatively new PTM that plays a role in inflammation and other cellular processes that are characterised by increased glycolysis and a break of the TCA cycle. Metabolites and metabolic enzymes are therefore increasingly discussed as interesting therapeutical targets.

## Abbreviations

ACOX1: acyl-coenzyme A oxidase 1
ADMSCs: adipose-derived mesenchymal stem cells
Akt: protein kinase B
ATP: adenosine triphosphate
CBP: cyclic adenosine monophosphate response element binding protein
cccDNA: covalently closed circular DNA
CoA: coenzyme A
CPS1: carbamoyl phosphate synthase 1
CPT1A: carnitine palmitoyl transferase 1A
CS: citrate synthase
DCs: dendritic cells
ECHA: mitochondrial trifunctional enzyme subunit alpha
ENO1: enolase-1
FBN1: fibrillin 1
GC: gastric cancer
GLS: glutaminase
GPR91: G protein-coupled receptor 91
GSH: glutathione
HAT1: histone acetyltransferase 1
HBV: hepatitis B virus
HCC: hepatocellular carcinoma
HIF-1α: hypoxia-inducible factor 1alpha
H_2_O_2_: hydrogen peroxide
IDH: isocitrate dehydrogenase
IL-1β: interleukin-1beta
KAT2A: lysine acetyltransferase 2A
KO: knockout
LDHA: lactate dehydrogenase A
LPS: lipopolysaccharide
MAVS: mitochondrial antiviral signalling protein
MDH2: malate dehydrogenase 2
MS: mass spectrometry
NAD^+^: nicotinamide adenine dinucleotide
NADH: reduced form of nicotinamide adenine dinucleotide
NADPH: reduced form of nicotinamide adenine dinucleotide phosphate
NSP14: nonstructural protein
OGDH: 2-oxoglutarate dehydrogenase E1
OTC: ornithine transcarboxylase
OxPhos: oxidative phosphorylation
PDAC: pancreatic ductal adenocarcinoma
PGK: phosphoglycerate kinase
PHD: prolyl hydroxylase
PI3K: phosphatidylinositol-3 kinase
PKM2: pyruvate kinase M2
PTMs: post-translational modifications
ROS: reactive oxygen species
SAH: subarachnoid haemorrhage
SCL: succinyl coenzyme A ligase
SDH: succinate dehydrogenase
SDHA: succinate dehydrogenase subunit A
SHMT2: serine hydroxymethyltransferase-2
SIRT 5: sirtuin 5
SOD1: superoxide dismutase 1
Succ: succinate
SUCLA2: succinate-coenzyme A ligase adenosine diphosphate-forming subunit beta
TAGLN2: transgelin-2
TCA: tricarboxylic acid
TPI: triosephosphate isomerase
UCP-1: uncoupling protein-1
VDAC: voltage-dependent anion channel
VLCAD: very long-chain acyl-coenzyme A dehydrogenase
WT: wild-type
α-KGDH: alpha-ketoglutarate dehydrogenase

## Declarations

### Copyright

© The Author(s) 2023.
